# Hyaluronan-Based Hydrogel Scaffolds for Limbal Stem Cell Transplantation: A Review

**DOI:** 10.3390/cells8030245

**Published:** 2019-03-14

**Authors:** Mazyar Yazdani, Aboulghassem Shahdadfar, Catherine Joan Jackson, Tor Paaske Utheim

**Affiliations:** 1 Department of Medical Biochemistry, Oslo University Hospital, Ullevål, 0450 Oslo, Norway; catherinejoanjackson@gmail.com (C.J.J.); utheim2@gmail.com (T.P.U.); 2 Center for Eye Research, Department of Ophthalmology, Oslo University Hospital, Ullevål, 0450 Oslo, Norway; aboulghassem.shahdadfar@medisin.uio.no; 3 Department of Plastic and Reconstructive Surgery, Oslo University Hospital, 0450 Oslo, Norway; 4 Institute of Oral Biology, Faculty of Dentistry, University of Oslo, 0318 Oslo, Norway; 5 Department of Maxillofacial Surgery, Oslo University Hospital, 0450 Oslo, Norway; 6 Department of Ophthalmology, Vestre Viken Hospital Trust, 3019 Drammen, Norway; 7 Department of Ophthalmology, Stavanger University Hospital, 4011 Stavanger, Norway; 8 Department of Clinical Medicine, Faculty of Medicine, University of Bergen, 5020 Bergen, Norway; 9 Department of Ophthalmology, Sørlandet Hospital Arendal, 4604 Arendal, Norway; 10 National Centre for Optics, Vision and Eye Care, Faculty of Health Sciences, University of South Eastern Norway, 3603 Kongsberg, Norway

**Keywords:** hyaluronic acid, hyaluronan, hydrogel scaffolds, stem cell-based therapy, transplantation, limbal stem cell deficiency (LSCD)

## Abstract

Hyaluronan (HA), also termed hyaluronic acid or hyaluronate, is a major component of the extracellular matrix. This non-sulfated glycosaminoglycan plays a key role in cell proliferation, growth, survival, polarization, and differentiation. The diverse biological roles of HA are linked to the combination of HA’s physicochemical properties and HA-binding proteins. These unique characteristics have encouraged the application of HA-based hydrogel scaffolds for stem cell-based therapy, a successful method in the treatment of limbal stem cell deficiency (LSCD). This condition occurs following direct damage to limbal stem cells and/or changes in the limbal stem cell niche microenvironment due to intrinsic and extrinsic insults. This paper reviews the physical properties, synthesis, and degradation of HA. In addition, the interaction of HA with other extracellular matrix (ECM) components and receptor proteins are discussed. Finally, studies employing HA-based hydrogel scaffolds in the treatment of LSCD are reviewed.

## 1. Introduction

Karl Meyer and John Palmer were the first to extract hyaluronic acid (conjugate base hyaluronate) from bovine vitreous humor in 1934. They named it based on its appearance (hyalos: glass in Greek) and structure (uronic acid: one of sugar molecules) [1]. Endre Balazs later defined various forms of the molecule in 1986. The suggestion of “hyaluronan” (HA) by Balazs (1986) was found to fit well with the international nomenclature of polysaccharides and was subsequently adopted [2].

HA, a well-conserved biopolymer with a disaccharide repeat unit in mammalian species, is one of the main components of the extracellular matrix (ECM) with widespread presence in many tissues in the body. Its concentration varies widely in different tissues: e.g., HA concentration is much lower in lung ECM (15–150 µg/g) and the vitreous humor of the eye (200 µg/g) compared to in the ECM of skin (500 µg/g) and synovial fluid (1400–3600 µg/g). Hyaluronan synthases (HASs) are its natural synthesizer in cells, which produce linear hyaluronan polymers with different chain lengths ranging from small to large molecules [1,3,4,5].

HA binds either to other hydrophilic molecules or to water. The latter forms a viscoelastic stiff substance that provides a unique structure, facilitating specialized functions in tissues (e.g., lubrication, hydration, and water transport) and cells (e.g., motility, adhesion, and organization). The biocompatibility, biodegradability, bioactivity, non-immunogenicity, and non-toxicity of HA promote its potential use in a variety of clinical applications. One fast-growing area of use employs HA-based polymers in tissue culture scaffolds that facilitate transplantation and the process of regeneration [2,6,7,8]. 

Tissue culture scaffolds, also termed synthetic extracellular matrices (ECMs), are designed as temporary supports that mimic the in vivo microenvironment or niche ECM. Scaffolds can be used clinically in the form of an acellular implant to stimulate cellular ingrowth and de novo tissue synthesis, to deliver required growth factors or to carry a cell type of interest (or its component) previously expanded in vitro. In the latter case, the propagated cells can be differentiated or undifferentiated (stem cells). The composition of the scaffold affects cell phenotype through the provision of physical and biochemical cues that maintain cell morphology, behavior, and responsiveness. In this regard, hydrogel scaffolds have been shown to be an attractive choice over other available HA-based forms (e.g., meshes and sponges) [9,10,11,12]. 

The HA hydrogel is a 3D network of polymer–polymer and hydrophilic polymer–water molecular interactions. Its physical properties, such as viscosity, elasticity, stiffness, shape, and structure, can be transformed by chemical modification [13,14]. Such flexibility in tailoring HA-based hydrogel polymers make them good candidates for synthesis of tissue culture scaffolding for transplantation of stem cells [15], a successful method used in the treatment of limbal stem cell deficiency (LSCD) [16]. This condition occurs following loss of functional limbal stem cells caused by direct damage and/or changes in their local microenvironment [17,18]. New studies providing insight into action mechanisms of modified HA matrices and discovering advantages in using them for treating LSCD may further encourage their clinical applications. For example, a recent study by Gesteira et al. [19] showed the importance of the HA microenvironment in maintenance of the limbal stem cell phenotype. The authors reported that differentiation of stem cells into corneal epithelial cells is dependent on the distance of limbal stem cells from the HA-rich niche during outmigration. 

This study reviews the physical properties, synthesis, and degradation of HA. In addition, the interactions of HA with other ECM components as well as cell receptor proteins are discussed. Finally, the use of HA-based hydrogel scaffolds in the treatment of LSCD is reviewed. 

## 2. Structure, Size, Synthesis, and Degradation of Hyaluronan

**Figure 1:**
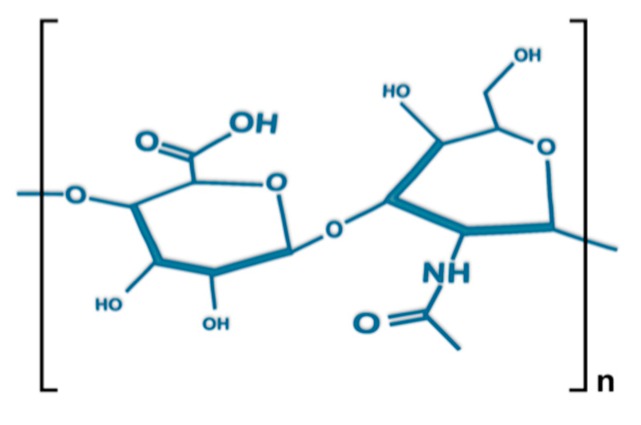
Structure of the disaccharide (d-glucuronic acid and *N*-acetyl-d-glucosamine) repeat unit of hyaluronic acid (HA).

The molecular formula of HA, a non-sulfated glycosaminoglycan (GAG), is (C_14_H_21_NO_11_)_n_, where one d-glucuronic acid bonds with one *N*-acetyl-d-glucosamine by glycosidic linkage between β-1,4 and β-1,3 (Figure 1). The synthesized bio-polysaccharides formed by these repeating building blocks differ in chain length and can create GAGs several million daltons in size (e.g., 8000 kDa in vitreous). HA chains present a semi-flexible random structure with a typical length of ~5 nm. The low-density chain segments are able to expand in aqueous solutions, forming large hydrodynamic domains. This unique characteristic is a product of the high molecular weight of HA, with the large size of its monomers causing local stiffness, hampered rotations near its glycosidic linkages, and continual formation/breaking of inter-residue hydrogen bonds [20,21,22,23,24]. 

The configuration and role of HA is determined by its size. This particularly affects its role in the ECM, where it provides structural and biochemical support to surrounding cells. HA can combine with other molecules, such as growth factors and cytokines. Hence, any structural change in the HA polymer can influence the extracellular microenvironment. High conservation of the structural features of HA across mammalian species illustrates the important role of HA in facilitating normal cellular function [3,7,8,25]. 

Two forms of HA have been classified based on size: low molecular weight (~200 kDa) and high molecular weight (~2000 kDa). Despite being composed of similar subunits they exhibit different biological activities. For instance, it has been proposed that high-molecular-weight HA is involved in reducing inflammatory responses, whereas low-molecular-weight HA induces expression of inflammatory mediators [19,26,27,28].

Synthesis of HA chains in vertebrates is performed by a class of integral membrane proteins, HASs (HAS1, 2, and 3). These enzymes are site-specific and they distinguish HA from other GAGs [29]. Following HA synthesis, HAS enzymes help to extrude these space-occupying polymers into the extracellular space instead of letting them accumulate in cells [30]. High-molecular-weight HA is thought to be synthesized by HAS1 and HAS3 and low-molecular-weight HA by HAS2 [19,31,32]. 

The expression of particular HAS enzyme isoforms is controlled by the combination of genetic and environmental factors. In the latter case, for example, it was shown that the expression of the three HAS isoforms is stimulus-dependent [33]. The authors reported a transcriptional study in normal human lung fibroblasts (NHLFs; 2801-1), foreskin fibroblasts (AG 1519), mesothelial cell lines (Mero-14), and lung glioma cell lines (U-118 MG) in response to various growth factors: fetal calf serum (FCS), platelet-derived growth factor-BB (PDGF-BB), transforming growth factor-β1 (TGFβ1), and phorbol 12-myristate 13-acetate (PMA). They showed the expression of all three HAS isoforms in mesothelial cells, only HAS3 in mesothelioma cells, and HAS2 and HAS3 in both lung fibroblasts and glioma cell line. Moreover, mesothelial cells expressed the maximum level of HAS2 after 6 h of exposure to PDGF-BB, whereas two other isoforms were slightly induced. The expression of HAS2 was slightly reduced following 6 h exposure to TGFβ1 but was strongly reduced in the presence of hydrocortisone. However, there was no significant expression level of HAS1 and HAS3. 

The half-life of HA in the body is short with a turnover time of hours to days, depending on the tissue. It is estimated that 5 g of a total 15 g HA in a 70 kg human is replaced per day. Enzymatic degradation of HA is mediated by hyaluronidases (there are six types in humans) through hydrolysis of disaccharides at hexosaminidic β (1,4) linkages. Epigenetic modifications are shown to be involved in the catabolism of HA [34]. For instance, Lokeshwar et al. [35] reported a strong influence on the expression of hyaluronidase 1 through methylation of its promoter at SP1/EGR1 binding sites. 

Enzymatic degradation occurs from both ends towards the center of the molecule, resulting in small HA fragments of various sizes. Non-enzymatic degradation of HA in the cell is conducted by oxidative stress processes, such as the generation of reactive oxygen species [28,36,37,38,39,40]. The main difference between these two catabolic mechanisms is their respective reaction products. Enzymatic action occurs at specific cleavage sites leaving chemically identical ends, whereas free radical catabolism leaves random breakage with oxidized termini. The cellular response to such diverse metabolic HA products might be different, although this has so far not been investigated [30].

The levels of HA in the body are regulated by the interplay of synthesis and degradation [41]. The dynamic balance between these two interactions along with the widespread presence of HA in the body, which requires a notable amount of energy, indicates the importance of HA, both structurally and functionally, for biological systems during evolution.

In addition to biological synthesis, HA can be derived in the laboratory from synthetic materials. Advantages of laboratory synthesis include minimal batch-to-batch variation as well as low risk of endotoxin and/or pathogenic contamination. Synthesis addresses the rising demand for HA. There has been a growing interest in developing laboratory HA synthesis protocols because chemical modification is feasible for making it suitable to use in a variety of purposes, ranging from tissue culture scaffolds to cosmetic materials [10,42,43].

## 3. Interactions of HA with ECM Components and Cell Receptor Proteins

The ECM is the non-cellular portion of all mammalian tissues. It is responsible for physical scaffolding of cells and initiates biochemical and biomechanical processes that mediate morphogenesis, proliferation, differentiation, and homeostasis of tissues. These structural and functional roles are made possible by the inherent properties of ECM, concomitant with its direct interaction with cell surface receptors, as well as local binding and release of growth factors. The structure of the ECM is highly dynamic, and its normal function is dependent on enzymatic and non-enzymatic remodeling [44,45,46,47].

**Figure 2:**
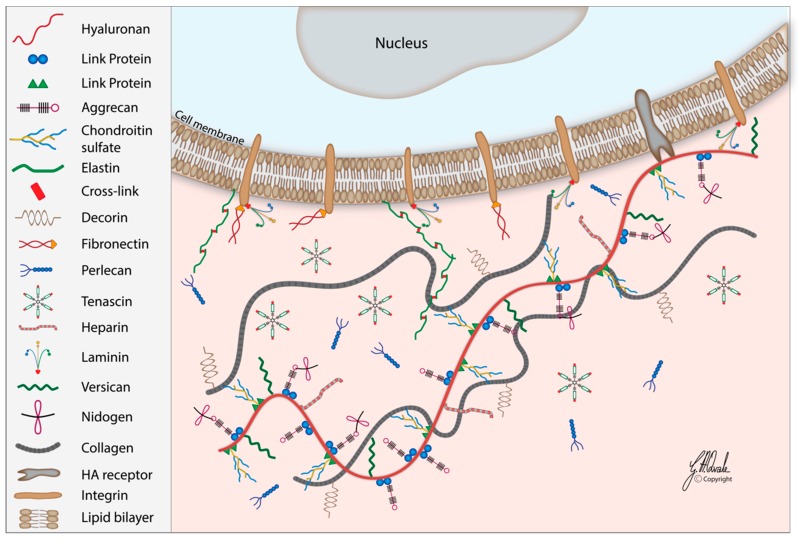
Schematic structure and interactions between some components of the extracellular matrix. Proteoglycans (e.g., versican, aggrecan, and decorin) and glycosylated proteins form strong covalent bonds between anionic amino acids and glycosaminoglycans (GAGs) (e.g., hyaluronan, chondroitin sulfate, and heparin) through link proteins. Glycoproteins (e.g., elastin, laminins, fibronectins, and tenascins) link structural molecules between each other as well as structural molecules and cells. Collagens, major insoluble fibrous proteins in the extracellular matrix (ECM), associate with other molecules, especially elastin.

The composition and topology of each ECM is uniquely tailored to tissue type. The main components are water, minerals, polysaccharides, and proteins [44]. The total number of core ECM and ECM-associated proteins (termed the core matrisome) in mammals is ~300, namely collagen subunits (43), proteoglycans (≥36; e.g., perlecan, versican, aggrecan, decorin) and glycoproteins (~200; e.g., elastin, laminins, fibronectins, thrombospondins, tenascins, nidogen) [48]. Proteoglycans are structural elements in the ECM that support cells, provide tissue turgor, and mediate development. They are glycosylated proteins with strong covalent bonds between anionic GAGs and amino acids. The main function of proteoglycans relies on the GAG component of the molecule [49,50]. Among the six major classes of GAGs in mammals, five are sulfated (keratin sulfate, dermatan sulfate, chondroitin sulfate, heparan sulfate, and heparin) and covalently linked to proteins. The final class, HA, is unsulfated and its aggregated form is caused by non-covalent interaction between individual proteoglycan link proteins (Figure 2) [51,52]. The diverse molecular weight of HA regulates various cell signaling pathways though binding and activation of specific cell surface receptors, but its wide range of roles is attributed to the large number of HA-binding proteins, known as hyaladherins. They differ in their affinity, specificity, intracellular localization, expression, and regulation in different tissue types [41,53].

Many hyaladherins, such as cluster of differentiation 44 (CD44), lymphatic vessel endothelial HA receptor-1 (LYVE-1), tumor necrosis factor-stimulated gene-6 (TSG-6), aggrecan, brevican, neurocan, and versican belong to a superfamily of link modules (proteoglycan tandem repeats). Structurally, they have a common domain of approximately 100 amino acids, forming two α-helices and two triple-stranded antiparallel β-sheets that facilitate the ligand-binding process [53,54,55]. The other category of HA-binding proteins contains dissimilar domains with structural differences in their primary sequence. For instance, a murine isoform of hyaluronan-mediated motility (RHAMMv4), a member of the non-link module hyaladherins (NLMH) family, includes a 62-amino acid segment that forms a helix–loop–helix motif. The NLMH category comprises a diverse group of proteins, which is still expanding in number, e.g., CD38, integral membrane protein (IMP)-150 and sialoproteoglycan associated with cones and rods (SPACRCAN) [53,56].

## 4. Challenges in Designing HA-Hydrogel Scaffolds

HA must first be chemically modified before it can be used to synthesize HA-hydrogel scaffolds for use in regenerative medicine. The main functional groups targeted for this purpose are glucuronic acid, carboxylic acid, primary and secondary hydroxyl groups, and the *N*-acetyl group after deamination [14,57]. Depending on the type of alteration, the resulting derivatives are widely different in their properties. The modifications may result in unwanted as well as beneficial changes in biological function. For instance, enzymatic degradation of HA may be affected after chemical alteration by switching from non-inflammatory high-molecular-weight HA to pro-inflammatory low-molecular-weight HA in broken fragments [58,59]. Thus, parameters such as the source and concentration of HA, nature of the crosslinker, ratio of HA to crosslinker, and the buffer environment should be considered when designing HA-hydrogel scaffolds. Of these factors, the purity of HA and biosafety of the crosslinker are considered critical in clinical applications [10,60].

## 5. Stem Cell Deficiency in the Cornea

**Figure 3:**
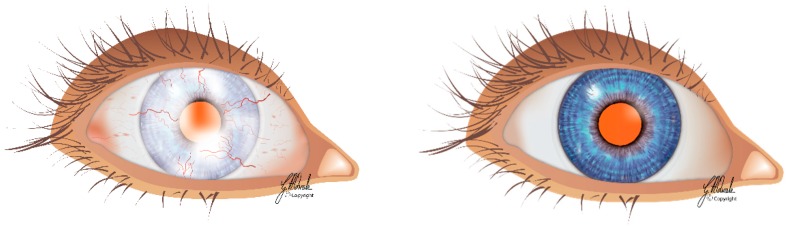
Schematic representation of limbal stem cell-deficient (left) and normal (right) eyes.

The cornea is the transparent part of the ocular surface. The clarity of the cornea is essential to facilitate vision by allowing transmission of light rays to the retina [61]. Homeostasis and regeneration of the corneal epithelium after minor injury are processes that depend on replacement cells provided by local limbal stem cell pools [62]. These cells are thought to be located in the periphery of the cornea known as the limbus. The self-renewal and proliferative capabilities of limbal stem cells enable the limbus to function as a barrier to ingrowth of the conjunctival epithelium [63]. The reduction in number or impairment in function of limbal stem cells may result in a condition known as LSCD (Figure 3) [18]. It could be caused by direct damage to the limbal stem cells and/or changes in their microenvironment due to intrinsic and extrinsic insults [17,64].

The etiology of LSCD can be classified as primary/hereditary (e.g., aniridia, dyskeratosis congenital, and neurotrophic keratopathy) and secondary/acquired (e.g., inflammatory ocular surface disease, and chemical and thermal burns) [65]. This condition is normally characterized by ingrowth of conjunctiva, photophobia, irritation, pain, blepharospasm, epiphora, severe visual impairment, and corneal blindness [63,65].

Additional mechanisms leading to LSCD include mutations in *PAX6*, a regulator of transcription with key role in the development of neural tissues, particularly the eye [66]. Other possible mechanisms are linked to growth factors involved in limbal stem cell modulation (e.g., EGF, IGF, FGF) and genes involved in the induction of differentiation, stratification, and maintenance (e.g., *CXCR4*, *DKK2*, and *DKK4*) [65,67,68,69,70].

## 6. Stem Cell-Based Therapy for Treatment of LSCD

The choice of treatment and rate of success for patients with LSCD depends on several factors, including the cause of the condition, size of the limbal injury (partial or total), number of eyes affected (unilateral or bilateral), and status of adjacent tissues (conjunctiva and eyelid). The ocular surface must also be prepared before treatment. Treatment options thus range from scraping off conjunctival epithelium covering the corneal surface in the case of early-stage partial LSCD to combining this mechanical debridement with stem cell-based therapy for total LSCD [71].

Treatment of LSCD aims to restore or replace normal functioning limbal stem cells to promote regeneration of the corneal epithelium. Traditionally, autologous (in the case of unilateral) or allogeneic (in the case of bilateral) grafts were the methods of choice. The ex vivo expansion of limbal stem cells from the healthy eye, as well as other potential sites for biopsy explant tissue, have been used for the production of autologous cell sheets on a scaffold to treat unilateral and bilateral LSCD. Alternate sites for autologous biopsy harvest include oral mucosal, epidermal, embryonic, umbilical cord, hair follicle bulge, immature dental pulp, and orbital fat-derived and bone marrow-derived mesenchymal stem cells [65,72]. It is believed that the transplanted cells create a suitable microenvironment for self-regeneration of the corneal epithelium from remaining dormant endogenous stem cells. Additionally, transplanted stem cells may supply the limbus with new cells [16,64]. A study of cell survival following transplantation by Daya et al. [73] showed the presence of DNA from transplanted limbal epithelial cell sheets on the ocular surface 9 months after operation, whereas others have reported the presence of donor cells 12 weeks to 3.5 years later [74,75,76,77].

## 7. HA-Based Hydrogel Scaffolds in the Treatment of LSCD

The most common cell carrier/support scaffold used in ocular surface reconstruction is the amniotic membrane. The drawbacks of limited transparency and mechanical strength, risk of disease transmission, poor standardization of preparation, and biological variability have encouraged the development of alternative membranes [78,79]. Scaffolds used, to date, include several natural and synthetic polymer scaffolds such as fibrin, siloxane hydrogel contact lenses, human anterior lens capsules, collagen, plastic compressed collagen, crosslinked collagen, synthetic and natural fiber electrospun scaffolds and magnetically oriented scaffolds [80]. Studies indicating the biocompatibility of HA with human corneal epithelial cells (hCECs) suggest that HA-based hydrogel scaffolds may be suitable carriers for delivery of hCEC [81,82].

Fiorica et al. [83] were the first to use HA hydrogels chemically crosslinked with a polyaspartamide derivative (PHEA-EDA) as a substitute for the amniotic membrane for delivery of human epithelial limbal cells. Their results support the potential clinical application of HA/PHEA-EDA hydrogels in the treatment of corneal damage as a carrier scaffold for transplantation of limbal stem cells. Thereafter, Kiiskinen [84] investigated co-culture of hCECs and human adipose stem cells (HASCs) in a 3D HA hydrogel with or without collagen type I from either human or rat origin. They showed that HASCs have an enhancing effect on the growth and differentiation of the co-cultured hCECs. Moreover, cell survival was better in the absence of collagen. This study suggested HA-hydrogel scaffolds could be used as a potential carrier for future applications in ocular surface reconstruction. Later, Chen et al. [85] developed a HA-hydrogel scaffold for ex vivo culture of limbal stem cells in a xeno-free culture system. They used a commercially available HyStem^®^-C hydrogel kit including three main components: thiol-modified hyaluronan (gycosil^®^), thiol-reactive polyethylene glycol diacrylate (PEGDA) crosslinker (extralink^®^), and thiol-modified collagen (gelin-S^®^). Their developed culture system excluded the risk of xeno-component contamination during expansion of regenerative limbal stem cells. The regenerated epithelium presented a similar characteristic phenotypic profile compared to that seen in limbal stem cells in vivo.

In spite of several studies on the physical and chemical properties of HA-hydrogel scaffolds [86,87,88] and their extensive application in regenerative medicine (e.g., chondrogenesis, osteogenesis, adipogenesis, and muscular regeneration using multipotent stromal cells) [14,15,36,89], since its discovery in 1934, not enough attention has been paid to their use as ophthalmic biomaterials [90,91,92]. Recent findings indicating a HA-rich microenvironment in the limbal stem cell niche [19], as well as the association between HA and several functions of limbal stem cell and corneal keratocytes (e.g., adhesion, phenotypic expression, biosynthetic capacity, and differentiation) [19,93] encourage future research on the use of HA-hydrogel scaffolds in the treatment of LSCD.

## 8. Conclusions and Future Work

HA, a well-conserved biopolymer with a disaccharide repeat unit, is one of the main components of the ECM with widespread presence in many tissues. It plays a key role in normal cell proliferation, growth, survival, polarization, and differentiation. The cornea has a HA-specific matrix in the limbal stem cell niche and any disruption to its integrity could lead to local or systemic reactions, such as an increased inflammatory response. More research is needed to better characterize the precise structural composition of this matrix and identify the specific length of the HA chains found in the limbal niche.

HA properties and structure can be transformed by chemical modification, making its derivatives, such as hydrogels, good candidates for synthesis of tissue culture scaffolding for transplantation of stem cells. New studies providing insight into action mechanisms of modified HA matrices can help with designing more biocompatible hydrogel scaffolds and discovering their advantages for therapeutic transplants.

Recent studies using an in vitro single or co-culture system as 2D monolayer or 3D spheroid are promising and suggest potential applications of HA hydrogels as carrier scaffolds in the treatment of LSCD. Future research should therefore concentrate on the investigation of in vivo applications in ocular surface reconstruction, particularly limbal stem cell transplantation.
